# SARS-CoV-2 Risk Quantification Model and Validation Based on Large-Scale Dutch Test Events

**DOI:** 10.3390/ijerph19127238

**Published:** 2022-06-13

**Authors:** Bas Kolen, Laurens Znidarsic, Andreas Voss, Simon Donders, Iris Kamphorst, Maarten van Rijn, Dimitri Bonthuis, Merit Clocquet, Maarten Schram, Rutger Scharloo, Tim Boersma, Tim Stobernack, Pieter van Gelder

**Affiliations:** 1 Department Values, Technology and Innovation, Delft University of Technology, 2628 CD Delft, The Netherlands; laurensznidarsic@gmail.com (L.Z.); p.h.a.j.m.vangelder@tudelft.nl (P.v.G.); 2 HKV Lijn in Water, 8232 JN Lelystad, The Netherlands; 3 Radboudumc, 6525 GA Nijmegen, The Netherlands; vossandreas@gmail.com (A.V.); tim.stobernack@gmail.com (T.S.); 4 Canisius-Wilhelmina Hospital, 6532 SZ Nijmegen, The Netherlands; 5 Breda University of Applied Sciences, 4817 JS Breda, The Netherlands; donders.s@lcb.nu (S.D.); kamphorst.i@lcb.nu (I.K.); maarten@mbadvies.nl (M.v.R.); 6 Fieldlab Program Committee, 1507 CC Zaandam, The Netherlands; dimitri.bonthuis@fieldlabevenementen.nl (D.B.); merit@sportinnovator.nl (M.C.); maarten.schram@fieldlabevenementen.nl (M.S.); rutger.scharloo@fieldlabevenementen.nl (R.S.); tim.boersma@bfcc.nl (T.B.)

**Keywords:** COVID-19, infection risk, events

## Abstract

In response to the outbreak of SARS-CoV-2, many governments decided in 2020 to impose lockdowns on societies. Although the package of measures that constitute such lockdowns differs between countries, it is a general rule that contact between people, especially in large groups of people, is avoided or prohibited. The main reasoning behind these measures is to prevent healthcare systems from becoming overloaded. As of 2021 vaccines against SARS-CoV-2 are available, but these do not guarantee 100% risk reduction and it will take a while for the world to reach a sufficient immune status. This raises the question of whether and under which conditions events like theater shows, conferences, professional sports events, concerts, and festivals can be organized. The current paper presents a COVID-19 risk quantification method for (large-scale) events. This method can be applied to events to define an alternative package of measures replacing generic social distancing.

## 1. Introduction

In response to the SARS-CoV-2 pandemic, many governments implemented measures to reduce the risk of infections. Social distancing to reduce the number of contacts is the main measure to reduce the transmission of SARS-CoV-2 [1]. To support social distancing, governments all over the world have taken measures, resulting in various types of (partial) lockdowns to reduce the number of contacts between people and limit gathering in groups. At the same time, Dutch event organizers affirmed that organizing events on the basis of social distancing would be economically detrimental.

### 1.1. Research Question

The aim of this research was to develop a model to determine the risk for infection at events during the pandemic, and the effectiveness of alternative measures instead of generic social distancing at these events. During test events, we evaluated several measures and determined how the risk for infection at these events compared to the average risk of infection during a lockdown or the risk at different locations. In this study we distinguish between four types of events:

1. Type I: Indoor, passive (theater show or conference),
2. Type II: Indoor, active (concert or dance events),
3. Type III: Outdoor, active (public sports events),
4. Type IV: Outdoor, active festival (festivals).

Regular contact matrices distinguish describe the number of contacts between people at a certain location between classes of age of the people at a location. The most recent contact matrix for The Netherlands distinguishes the locations of home, school, work, and other, and the number of contacts is based on the pre-pandemic situation [2]. This class “other” contains multiple locations which might contribute very differently to the risk for infections. Standard social contact data [2,3] also do not apply because of governmental measures and personal behavior changes. We have therefore gathered actual data regarding contacts and settings representing peoples’ whereabouts during the pandemic, as well as statistics regarding the epidemiologic situation during the pandemic; they have been used to develop a causal risk quantification model.

In this study, we have developed a causal model to describe the risk for infections at these events. Causal modeling forms an alternative for physical or biological models, and (as such) can support the insight into the interdependencies between the constituent parts of complex systems as these events [4]. A causal risk model is developed based on available data on infections and contacts among people during the pandemic. This risk model determines the risk for infection per hour at events depending on the type and design of the event (as the number of people, pre-testing, crowd control, ventilation, etc.) the circumstances of the event given the epidemiologic situation at that moment (as prevalence, vaccinations or recent infections, variant of the virus, etc.).

### 1.2. The Infection Risk per Hour

Most of the available literature estimates the reproduction number R0 of Severe acute respiratory syndrome coronavirus 2 (SARS-CoV-2) is between 2 and 4 [5,6]. For Western Europe, the R_0_ is estimated at 2.2 [7] and the Dutch National Institute for Public Health and the Environment (RIVM) estimated a range between 2 and 3 [8]. The probability of an infected person infecting another person peaks during the first week of illness, after which this probability drops significantly [9]. Based on data from Wuhan, the incubation time was on average 5.2 days and at least 4 days [10]. Later studies based on more data indicated that the average incubation time ranged between 5.2 and 6.65 days and could be up to 14 days [11]. We decided to express the probability of infection during the period of exposure during an event as the probability per hour. This risk can be compared to the infection risk outside these events during the pandemic. When R_0_ is 2.2, people are infectious for 7 days and a prevalence of 0.77% in the Netherlands this means that the probability that a person infects another person is 1.04 × 10^−4^ per hour.

The number of positive SARS-CoV-2 tests in the Netherlands is reported by the RIVM on a weekly basis [12]. These reports include the expected location where people had been infected. Examples of these locations are homes, work, home while having visitors (friends or family), leisure, schools, catering industry, elderly houses, and events. These numbers were based on the positive tests and surveys among people who tested positive taken by the regional health services, the results were daily reported to the RIVM by the regional health offices. The measures taken by the government strongly influence the number of contacts at these locations. Although of all location types most people were infected at home, the probability per hour for a person of being infected at this type of location was relatively small compared to other types.

We conducted a survey to estimate the time people spend at several locations and the number of contacts at these locations. The individual probability per hour of being infected for each location can be compared when the absolute numbers of infections are corrected for the duration of the stay at a location and the number of people at these locations. These figures can be used as benchmarks when deciding whether to have events.

## 2. Methodology: SARS-CoV-2 Risk Quantification Model

### 2.1. Methodology

In this study, a SARS-CoV-2 risk quantification model was developed based on datasets collected by the National Institute for Public Health and the Environment (RIVM) and municipal health services (GGD) collected datasets during the SARS-CoV-2 pandemic.

The average risk of an infection *R* at the event of type *i* per hour is defined as the combination of the risk of different groups of people infecting each other:

$$R_{i}=R_{i,a}+R_{i,b}+R_{i,c}+R_{i,d}\;in\;which\;R_{i,a}=\left(1-F_{M,i}\right)(C_{1,i}A_{1}+(C_{2,i}A_{2}\left(1-F_{Y,x})\right)P\;Z\left(1-F_{T,1}\right)V_{1\;}V_{1}\left(1-R_{1}^{I}\right)\left(1-R_{1}^{S}\right)\;R_{i,b}=\left(1-F_{M,i}\right)(C_{1,i}A_{1}+(C_{2,i}A_{2}\left(1-F_{Y,x})\right)P\;Z\left(1-F_{T,1}\right)V_{1\;}V_{1}\left(1-R_{1}^{I}\right)\left(1-R_{2}^{S}\right)\;R_{i,c}=\left(1-F_{M,i}\right)(C_{1,i}A_{1}+(C_{2,i}A_{2}\left(1-F_{Y,x})\right)P\;Z\left(1-F_{T,2}\right)\;V_{1}\;V_{1}\left(1-R_{2}^{I}\right)\left(1-R_{1}^{S}\right)\;R_{i,d}=\left(1-F_{M,i}\right)(C_{1,i}A_{1}+(C_{2,i}A_{2}\left(1-F_{Y,x})\right)P\;Z\left(1-F_{T,2}\right)\;V_{1\;}V_{1}\left(1-R_{2}^{I}\right)\left(1-R_{2}^{S}\right)$$

We consider:

1. Two types of individuals, *j*, who can attend an event and for which different test regimes can apply. *j* = 1 corresponds to unvaccinated individuals without a documented infection, *j* = 2 corresponds to vaccinated individuals or people with a documented infection. *V_j_* is the proportion of the people in each group. The sum of *V_1_* and *V_2_* is always 100%. At each event, we assume a homogeneous mixing of all people. Therefore, $R_{i,a}$ is the risk that a *j* = 1 individual infect a *j* = 1 individual, $R_{i,b}$ is the risk that a *j* = 1 individual infect a *j = 2* individual, $R_{i,c}$ is the risk that a *j* = 2 individual infects a *j* = 1 individual, and $R_{i,b}$ is the risk that a *j* = 2 individual infect a *j* = 2 individual.
2. The prevalence *P* describes the proportion of infectious people in the community during the event.
3. The complete role of virus-laden droplets and aerosol transmission is poorly understood [13]. We distinguish between two contact classes that are most significant: *C_1_* is the number of contacts per hour within 1.5 m (droplets) and *C_2_* is the number of contacts per hour within 10 m (aerosols). Smart logistics and crowd control at an event reduce contacts and help avoid gatherings of large groups of people. Therefore, specific data were collected at test events because the duration of events is limited the latency period is shorter than the duration of stay at each location.
4. Factor *F_T,j_* describes the effectiveness of testing for group *j* in a certain window prior to the event.
5. Vaccination and earlier infections result in a level of immunity and reduce the infectiousness *I* and susceptibility *S* of an individual. Because people without a documented infection might have an earlier infection the weighted *I* and *S* have to be defined for individuals of type *j*. $R_{j}^{I}$ and $R_{j}^{S}$ are the relative infectiousness and susceptibility for an individual of type *j* related to a naïve person. During the test events used to validate the model, all people were considered to be naïve (which means that they do not have any immunity), nobody was vaccinated, and a limited (not significant) number of people have had an earlier infection.
6. *F_Y,x_* is the percentage of reduction of aerosols because of ventilation. When *x* = 0, locations are ventilated according to building codes, *x* = 1 means very well ventilated and *x* = 2 is outdoor.
7. *F_M_* is the risk reduction factor for personal protection measures such as mouth-nose masks.
8. *Z* is a multiplier for variants that are more infectious than the Alpha variant which was dominant during the data collection for the transmission coefficients.

### 2.2. Transmission Coefficients A_1_ and A_2_

The parameters *A_1_* and *A_2_* are transmission coefficients for exposure to droplets and aerosols. We distinguished between two periods: 15 September–13 October 2020 and 14 October–15 December 2020. On 13 October additional measures were implemented by the Dutch Government, and on 15 December 2020, a new lockdown was implemented. For these two periods, we collected contact data of persons by a questionnaire and combined them with statistical data [14]. We applied linear regression via least square minimization (as the most common and proven approach for linear models) on a dataset of infections per location, duration, and contact per location. We used data for the locations at home, at work, visitors at home, and at leisure. The location at work includes healthcare workers. The weekly RIVM reports [13] (for example 15 December 2020) showed that about 16% of the positive tests of people between 18 and 69 years old were healthcare workers, only 11% of the people in this age group are healthcare workers. However, the same report mentions that healthcare workers were tested more frequently and earlier than non-healthcare workers, therefore the ratio of asymptomatic people among the positive tests will also be higher. Because no quantitative information was known about biases, we decided not to correct the data. For the other locations, the amount of available data was not sufficient. In the questionnaire, we asked people after:

1. The time spent at a certain location.
2. The number of persons in a range of 10 m.
3. The number of contacts within a distance of <0.5 m, between 0.5 and 1.5, and between 1.5 and 2.0 m for less than a minute, between 1 and 15 min, and more than 15 min.
4. The proportion of the time which was spent indoors, indoors and well ventilated or outdoors, given a type of location.

For each category of location, we assumed an average duration (30 s for the class <1 min, 8 min for contacts within 1 and 15 min, and 30 min for all contacts more than 15 min). For all indoor locations we assumed standard ventilated locations *F_y,0_* = 0, for well ventilated indoor *F_y,1_* = 0.5, for outdoor events *F_y,2_* = 1. All people were considered to be naïve, and testing was not available in this period.

This resulted in a probability per hour that a person will be infected given a contagious person at a distance of 1.5 m (*A_1_* = 6.376 × 10^−3^ per h) or 10 m (*A_2_* = 0.986 × 10^−3^ per h). Table 1 and Table 2 describe the data used for the least-squares regression.

Table 3 shows sensitivity analyses that the impact of other choices in the distances of 1.5 m and 10 m or for *F_y,x_* is limited (given a prevalence of 0.5%). For the reference situation, *C_1_* is 5 contacts per hour for the low contact event and 12.5 for the high contact event. *C_2_* is 10 contacts for the low contact event and 30 for the high contact event. The sensitivity analysis shows that the impact on the results sits within a bandwidth factor of 0.5.

### 2.3. Total Number of Infections at an Event

The expected number of infections $S_{\;}$ at event i is the combination of the product of $R_{i}\;$ and the duration (in hours) of the event $t_{i}$ and the number of people at the event $N_{i}$:

$$S_{i}=R_{i}t_{i}N_{i}$$

## 3. Validation of the Model

The model determines an average risk for infection. The data concerning SARS-CoV-2 infections gathered at the test events can be used to validate the model. During these tests, vaccinations were not yet available, and only a small amount of people had earlier infections. Therefore, all people were considered to be naïve, and all people who attended an event had to be tested ($V_{1}$ = 100%). The model outcome, an average number of infections, is based on a skewed probability distribution. For example, consider a large-scale event where 1000 people will join the event given a prevalence of 0.75%. Without pre-testing, 7.5 infectious persons would have attended the event, when *F_T,j_* = 0.95 on average 0.38 people would have been infectious at the event and could infect others. In the model we use the average number of contacts, there will be a probability distribution about these contacts some people will have many close contacts and others will see only a few people. Because of these uncertainties, it is expected that many events will be organized that will result in no or a limited number of infections, and a few events will result in many infections.

A model validation would need a large dataset during the SARS-CoV-2 pandemic. This large dataset is expected to cover the skewed probability distribution including events. Such a database, however, is not available. Data from media and the literature on superspreader events are biased as these always attract more attention. For loss of life modeling for natural hazards, the limited availability of data also leads to difficulties in conducting model validation. For example, the loss of life models for river and storm surge flooding as used in the Netherlands are based on the 1953 flood in Sealand and the flood caused by Hurricane Katrina in 2005 in the USA [15,16]. However, despite the limited validation, the model is still used to define the safety standards for Dutch levees which implies an investment program of multiple billions of EUR [17]. Although a perfect validation is not attainable, the available data can be used for a first validation of the model.

### 3.1. Internal Validation: Reproduction of Infections at Different Settings

**Figure 1:**
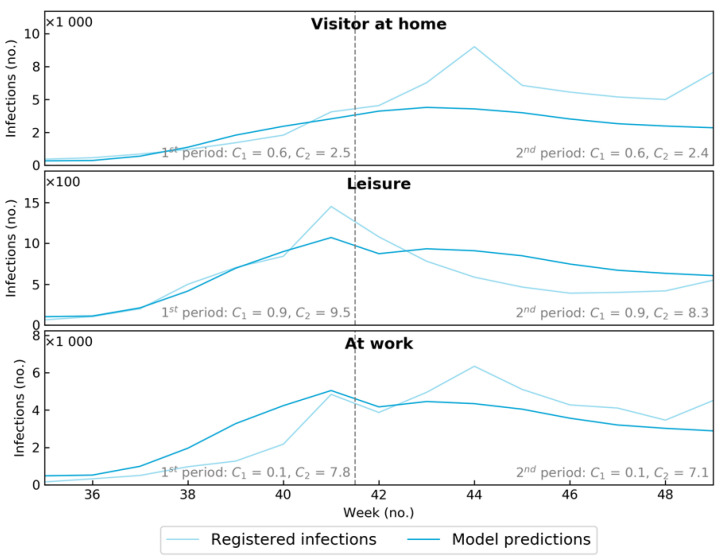
The number of infections based on internal validation by reproduction of infections at work, visitors at home, and at leisure.

First, the performance of the model can be checked by the reproduction of infections for the settings at work, visitors, and leisure time. Given the value for *A_1_* and *A_2_*, the prevalence as published by the RIVM and using data from the questionnaire for the type of locations “visitors at home”, “work”, and “leisure” to define *C_1_*, *C_2_*, *F_Y,x_* we estimated the number of infections by the model for these locations. These model results are compared with the measured amount of infection at these locations by RIVM and regional health services (see Figure 1). The infections “at work” are overestimated in the model, while infections in the setting of “at home with visitors” are underestimated. An explanation can be that people received more visitors than they admitted to in the survey or than allowed within the prevailing COVID-19-rules (two visitors per day until mid-October, and one per day afterward). Overall, we concluded that the outcomes support the results of the model. Room for improvement is available if more contact data are available.

### 3.2. External Validation: Test Events

The Fieldlab test events are for external validation. The test events occurred during the pandemic in the middle of a wave of infections. A lockdown was still in place and vaccination was not available yet. The input for the model is defined as:

During these test events, C_1_ and *C_2_* are measured for different variants of measures at these events and different types of events.

All Fieldlab participants and crew were asked to get tested on day five after the event, a request that was followed by more than 80% of the participants. In addition, all positive cases related to a Fieldlab event, identified by the regional Health Care Services, were included in the data set. Infections identified after an event included persons infected just before or after the pre-test or persons who had taken a PCR test around the cut-off of the PCR, thereby varying in outcomes. This means that people who were at the event and tested positive could also have been infected at other locations before or after the test event.

Very short, “passing” contacts of less than 10 s were not taken into account because these “passing contacts” are assumed not to be significant with regard to transmission. At the test events, the generic measures for social distancing were not in place. The risk of infection was reduced by several packages comprising variations in occupation rate, catering, crowd management, the use of masks, and ventilation protocols.

1. *P* is based on the prevalence of the date of the events as published by RIVM [13].
2. *V_1_* = 100%. Because vaccination was not available yet and earlier infections were very limited and not significant, all people were naïve. Only *j* = 1 individuals could attend the event and $R_{1}^{I}\;$ and $R_{1}^{S}\;$ therefore are equal to zero.
3. Persons with COVID-19-(like) symptoms were banned from participation. All visitors and crew needed a negative PCR test taken within 48 h before attending the event. As the PCR test may pick up low viral loads such as in cases of persons who recently recovered from COVID-19, the ratio of positive tests is higher than the ratio of asymptomatic people only [18], *F_T,1_* = 0.95.
4. *F_y,0_* = 0 if the ventilation meets the requirements of building codes. *F_y,1_* = 0.90 is applied when ventilation is significantly improved (with a CO2 value below 800 ppm). All venues have been checked prior to the event and during the event, the CO2 value was measured. Because all outdoor events were not completely open (the festival was in a tent, and football stadiums had a large roof), we assumed *F_y,2_* = *F_y,1_*. These values are based on expert judgment.
5. The effectivity of masks was estimated by medical experts. While the effectivity under in vitro conditions can be high [19], a low estimate is realistic for their effectiveness during in-vivo events because masks are not used correctly, and they may be ill-fitting. During all test events, different rules are applied. *F_M_* = 0.05 when the mask was used only when people were seated, and *F_M_* = 0.1 when the mask was used while people move (but not while they are drinking and eating). During type 2 and 4 events, *F_M_* was set at 0, as mask compliance was extremely low to non-existent.

The number (and risk of) infections at the event are estimated with the model based on the prevalence and measures at the test events. While in the post-event test results, the crew is also taken into account, the calculated risk for infections applies to visitors only. The model results can be compared with the confirmed infections after the events. In Table 4 the results of the test events have been summarized per type of event and compared to the model results. The detailed results of all events and measures are described elsewhere [20].

In the post-event tests, 14 cases have been identified where persons were possibly infected at the events. Other positive tests were excluded during interviews, for example, because participants had known close contacts with SARS-CoV-2 positive cases at their home or events around the same time. However, even in the 14 cases where participants were possibly infected, infections could have occurred in other places and situations at any time from around the pre-test, the day of the event, or even a few days after the event. If we use the Dutch average infection risk during the event as a control group we can estimate the number of infections that could be expected outside the event. The time spent at the event represents about 4–10% of the total time where people could be infected. If we also consider the risk at other locations, one or two of the possible infections could be (on average) related to the events.

During the eight test events, four persons were confirmed to be infected during the event. Confirmed infections are those infections that can be related to each other for example by sequencing or because of proven contacts with other positive tested people during the event. Two infections occurred while traveling home with a contagious person, which caused two infections at the type IV event. These are not infected at the event (and part of the model) but these are related to the event.

As the number of events was limited related to the expected probability distribution, the data analysis would have profited from a higher number of events. Still, the data can be used to check if the model is plausible. We believe that we included nearly all infected people at the event because we combined the information of regular testing procedures and the after-event tests. Overall, we concluded that the outcomes of the test events support the results of the model.

## 4. Discussion and Concluding Remarks

### 4.1. Reflection on the Validation of the Model

In the model, we assumed an average of infectious people in the Netherlands. Our assumptions were corroborated by the pre-event test results, which were in the range of the nationally reported prevalence of SARS-CoV-2. However, it might be that the prevalence among the visitors was higher than the assumed average. A first argument is that more young people attended the test events, and these age groups contribute relatively more than the elderly (as >60 years old) to the positive PCR tests [21]. A second argument is that the test events were held during the lockdown, and the non-risk-averse people who attend these events might also have attended more other activities. On the other hand, the susceptibility of younger naïve individuals is less than for elder people [22]. Because of these other activities, it can be expected that the source of infections for some of the cases identified after the event may be unrelated to the event.

On day 5 after the event, visitors and crew were tested, but our model exclusively calculates the risk for visitors, as contact data of the crew were not measured. During the events, the crew attempted to keep their distance from the participants and wore masks continuously.

In general, we found more PCR-positive people in the pre-event than in the post-event testing. As the PCR test may pick up low viral loads, some of the people testing positive, especially in the pretests group, may have recovered from COVID-19, consequently resulting in a higher positive test ratio. This in part explains the positivity rate in the pre- and post-event testing. However, in the after test, people with COVID-19 symptoms were also included. Only for (one of) the type IV events, the ratio of positive tests after the test was higher than in the pre-test. This corresponds to higher numbers of infections at the event.

A final remark about the model can be made regarding the data which is used to estimate *A*_1_ and *A*_2_. The contacts used to train the model were gathered during a period of a (partial) lockdown. Large-scale events were already prohibited or regulated. This could cause an underestimation of the risk in dynamic settings.

The model can be extended with the risk for loss of life and hospitalization using the relation with the age of people. However, once more data are available, this larger training set can help improve the model. Once more data of especially type IV and maybe type II events becomes available, the need for an additional factor for increased transmission at dynamic events can be identified and incorporated. We therefore recommend collecting more data about infections and events while the COVID-19 pandemic continues.

### 4.2. Final Conclusion and Recommendations

Based on the available but scarce information, the current risk quantification model results in plausible results, so the model cannot be rejected as being invalid.

The test events show that the Fieldlab measures at the events, which replace generic social distancing, reduce the risk for infections. This risk for infections can be compared to the risk in other settings at the same moment, or to a threshold that can be seen as an acceptable risk.

The risk for infections at an event depends on the type of event and the measures which are in place. The test events showed (depending on the type of event and the measures in place) that the risk of infection at these events can be reduced to a level equal to less than having a visitor at home or for some events the risk of getting infected at home situation.

We also recommend collecting more data to validate the model and analyze the impact of uncertainties in infections. We also recommend improving the dataset on which the transmission parameters are trained and to explore of more classes for distance and time are necessary. For new variants, impact on risk can be estimated by a multiplier (*Z)* and an update for the effectiveness of testing. Better insight can be made after an update of transmission coefficients *A_1_* and *A_2_* based on a dataset for these new variants; therefore, continuous data collection is needed.

### 4.3. Added Value for Decision Makers and Event Planners

Depending on the package of measures (such as testing, maximum occupation rate, ventilation) and the proportion of infectious people in the society the risk of infection at an event can be reduced. These measures can be an alternative for social distancing at these events which in general will lead to an increase in risk because of the increase in contacts per hour. The test events in the Netherlands showed that the risk at many of these events can be reduced to a level that is equal to the average risk people are exposed to outside these events. This means that events, with supporting measures, can be organized in such a way that they do not contribute more to the risk of infection per hour than other activities which are considered to be safer. When the total risk for infection in society, however, is too high, choices can be made regarding which activities have to be stopped. These decisions are outside the scope of our research.

**Figure 2:**
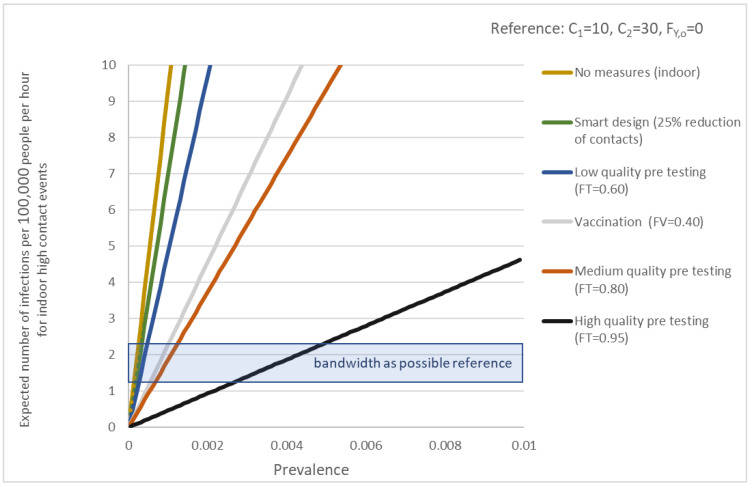
Risk of infections as a function of the prevalence for different packages of measures related to a possible reference.

To decide whether an event is accepted or decide the need for additional measures a threshold can be used. In Figure 2, such an example is given. The figure shows the expected risk of infections (per 100,000 people per hour) as a function of the prevalence. Figure 2 holds the same event but with different packages of measures. The horizontal bar is the threshold, now set at 1 or 2 infections per 100,000 people per hour. During the test events, the risk for infection for people at home was about 1 per 100,000 per hour, the average risk of all types of locations in the Dutch society was between 1 and 2 per 100,000 per hour. When the risk for an event is above the threshold additional measures are needed such as testing, smart design of the catering and crowd control, and occupation rates. These measures result in a reduction of the risk, when the risk is below the threshold the risk because of the event is acceptable. The developed COVID-19 risk quantification method for (large-scale) events can be applied to events to define an alternative package of measures replacing generic social distancing. The value for the thresholds is also a political choice. We therefore recommend a debate to discuss them in perspective to an acceptable risk for infection or load on the healthcare system.

## Figures and Tables

**Table 1:** Location and infection data used for the least-squares regression of *A_1_* and *A_2_* based on RIVM and the survey.

Setting	No. Positive PCR Tests (Source RIVM)	Hours at Location	Average Infections per Hour
At home	186,772	1.74 × 10^10^	1.08 × 10^−5^
Visitor	59,882	1.09 × 10^9^	5.49 × 10^−5^
Leisure	8530	1.49 × 10^8^	5.72 × 10^−5^
At work	46,881	1.00 × 10^9^	4.69 × 10^−5^

**Table 2:** Contact data used for the least-squares regression of *A_1_* and *A_2_* based on RIVM and survey data on the number of contacts per hour within a certain contact category, per setting.

	Between 2 and 1.5 m			Between 1.5 and 0.5 m			Less than 0.5 m					
	<1 min	1 < min < 15	>15 min	<1 min	1 < min < 15	>15 min	<1 min	1 < min < 15	>15 min	*C_1_*	*C_2_*	*F_y,x_*
Visitor	0.31	0.92	2.42	0.31	0.10	0.14	0.35	0.41	0.89	0.58	2.47	0.71
Leisure	1.28	0.49	3.18	1.11	0.06	0.00	1.13	0.06	1.72	0.89	8.88	0.53
At work	0.36	0.38	1.68	0.24	0.09	0.02	0.24	0.17	0.11	0.10	7.47	0.90

**Table 3:** Sensitivity analyses for ventilation and distance for contact classes. *F_V_*, *F_T_*, and *F_M_* are all 0.

Setting	Model	*C_1_*	*C_2_*	R Absolute *F_y,2_* = 100 and *F_y,1_* = 50	R Absolute *F_y,2_* = 95 and *F_y,1_* = 90	Difference
Low contact	Normal ventilated (reference)	5.0	30.0	3.07 × 10^−4^	3.07 × 10^−4^	0%
Low contact	Well ventilated	5.0	30.0	2.33 × 10^−4^	1.74 × 10^−4^	−25%
Low contact	Outdoor	5.0	30.0	1.59 × 10^−4^	1.67 × 10^−4^	5%
High contact	Normal ventilated (reference)	12.5	50.0	6.45 × 10^−4^	6.45 × 10^−4^	0%
High contact	Well ventilated	12.5	50.0	5.22 × 10^−4^	4.23 × 10^−4^	−19%
High contact	Outdoor	12.5	50.0	3.99 × 10^−4^	4.11 × 10^−4^	3%
Setting	Model	*C_1_*	*C_2_*	R absolute	*A_1_*, *A_2_* (×1000)	difference
Low contact (reference)	1.5 m for *C_1_*, 10 m for *C_2_*	5.0	30.0	3.07 × 10^−4^	6.38, 0.98	0.0%
Low contact	2.0 m for *C_1_*, 10 m for *C_2_*	8.9	30.0	2.21 × 10^−4^	2.71, 0.67	−28.1%
Low contact	1.5 m for *C_1_*, 8 m for *C_2_*	5.0	19.2	3.07 × 10^−4^	6.38, 1.54	0.0%
High contact (reference)	1.5 m for *C_1_*, 10 m for *C_2_*	12.5	50.0	6.45 × 10^−4^	6.38, 0.98	0.0%
High contact	2.0 m for *C_1_*, 10 m for *C_2_*	22.2	50.0	4.69 × 10^−4^	2.71, 0.67	−27.3%
High contact	1.5 m for *C_1_*, 8 m for *C_2_*	12.5	32.0	6.45 × 10^−4^	6.38, 1.54	0.0%

**Table 4:** Comparison model results with realization at test events of type i.

	Type I	Type II	Type III	Type IV
People at event (N) (without crew) (persons)	815	2341	1692	2960
Average duration (t) of event (hours)	4.4	4.3	3	7
Average prevalence (P) at events	0.0056	0.0061	0.0056	0.0077
Number of Pretests (incl crew) (persons)	1198	3078	2033	3890
Positive pre-test (persons)	11 (0.9%)	18 (0.6%)	12 (0.6%)	26 (0.7%)
After (5 d) tests (incl crew) (persons)	926	2603	1689	3168
Positive after test (persons)	1 (0.1%)	14 (0.5%)	4 (0.2%)	26 (0.8%)
Possible infections at event (realization) (persons)	0	4	0	12
Confirmed infections at event (realization) (persons)	0	0	0	4
Expected infections (S) at event (model) (persons)	0.04	0.28	0.05	0.54
Expected Infections (S) at event without measures (model) (persons)	0.86	5.56	1.14	10.81
Individual risk $(R_{i}$) per hour at event	1.12 × 10^−5^	2.62 × 10^−5^	1.00 × 10^−5^	2.61 × 10^−5^
Minimal individual risk per hour at event	1.4 × 10^−5^	1.5 × 10^−5^	8.04 × 10^−6^	1.6 × 10^−5^
Maximum individual risk per hour at event	8.5 × 10^−6^	4.3 × 10^−5^	1.6 × 10^−5^	3.6 × 10^−5^
Average individual risk (R home) per hour at home	1.06 × 10^−5^	1.14 × 10^−5^	1.06 × 10^−5^	1.45 × 10^−5^
Average individual risk (R visitor)per hour having a visitor	4.50 × 10^−5^	4.82 × 10^−5^	4.48 × 10^−5^	6.13 × 10^−5^
Individual risk per hour $(R_{i}$) at event without measures	1.25 × 10^−4^	5.10 × 10^−4^	2.24 × 10^−4^	5.22 × 10^−4^

## Data Availability

Data available on request. The data presented in this study are available on request from the corresponding author.
